# Women’s cancers in Sudan with a focus on cervical cancer: turmoil, geopolitics and opportunities

**DOI:** 10.3332/ecancer.2022.1433

**Published:** 2022-08-01

**Authors:** Nazik Elmalaika Husain, Amira Burhan, Iman A I Ahmed, Sulma I Mohammed, Nazik Hammad

**Affiliations:** 1 Department of Pathology, Faculty of Medicine and Health Sciences, Omdurman Islamic University, PO Box 382, 14415, Omdurman, Sudan; 2 Cervical Cancer Prevention & Research (CCPR) Unit, Department of Obstetrics & Gynaecology, Soba University Hospital, Khartoum, 13314, Sudan; 3 Department of Obstetrics & Gynaecology, Faculty of Medicine, University of Khartoum, Khartoum, 11115, Sudan; 4 Independent Global Health and Migration Expert, Greater Toronto Area, ON L5B 4N3, Canada; 5 Department of Comparative Pathobiology, Purdue University, West Lafayette, IN 47907, USA; 6 Purdue University Center for Cancer Research, Purdue University, West Lafayette, IN 47907, USA; 7 Division of Medical Oncology, Department of Oncology, Queen’s University, Kingston, ON K7L 5P9, Canada; a https://orcid.org/0000-0001-8333-5735; b https://orcid.org/0000-0002-2479-5675; c https://orcid.org/0000-0003-1617-3478; d https://orcid.org/0000-0002-6031-3442; e https://orcid.org/0000-0003-3963-5815

**Keywords:** Sudan, women’s cancer, female reproductive, gynaecology, health system, uterine cervix, HPV, challenges, policy, vaccine

## Abstract

Cancer is the leading cause of death worldwide and the second leading cause of death in Sudanese women. However, despite proven interventions for primary, secondary and tertiary prevention and the World Health Organization’s call to action toward eliminating cervical cancer, there has been little progress in addressing the cervical cancer burden in Sudan. This short communication intends to shed light on the challenges facing women’s cancers in Sudan, taking cervical cancer as an example. It also discusses the opportunities and suggests ways to improve the outcomes of women’s cancers in Sudan. Sudan’s government should urgently implement a broad public health strategy to improve outcomes for women with cancer. The cancer control plan should be aligned with international, evidence-based recommendations and adapted to local circumstances. It should strengthen health literacy, augment different health care interventions, including vaccination, committed screening programmes, early detection and proper diagnosis of symptomatic cases, a programmatic approach to active management and palliative care and ensure robust referral pathways. Policies are also needed in collaboration with the international community in addressing the cancer care needs of internally displaced and refugee women in Sudan. The strategy should consider overcoming the existing challenges and making the most opportunities available.

## Introduction

Cancer is the leading cause of death worldwide, with about 10 million individuals dying in 2020 alone [1]. Epidemiologists predicted that cancer incidence will increase to 28.4 million cases in 2040, a 47% increase compared to 2020, with a more significant increase in transitioning (64% to 95%) versus transitioned (32% to 56%) countries [1]. Although there is no accurate information on the disease burden in Sudan due to the lack of a functioning national cancer registry, GLOBOCAN estimated that cancer is the second leading cause of death in Sudanese people. In 2020, there were 27,382 new cancer cases and 17,055 cancer death [2].

The increasing cancer burden in Sudan poses tremendous challenges to the health system and the entire country’s economy due to lost productivity, premature death [3] and the high cost of cancer management. However, despite the increasing public awareness of cancer and its consequences in Sudan, the country is lagging in investing in cancer control programmes due to the instability of public health leadership, catastrophic spending on health with perpetually limited public financing, heavy reliance on curative at the expense of preventative care and over 79.4% out of pocket (OOP) health expenditure [4]. At the geopolitical level, this is enhanced by the continued civil unrest, corrupt regimes and consequent economic sanctions. Moreover, curative services for cancer face marked deficiency of laboratory and radio-diagnostic facilities, workforce shortages, lack of drugs and ever-shrinking radiotherapy facilities.

Sudan has almost no cancer prevention, screening or early detection programmes. Consequently, patients present with late-stage disease resulting in poor outcomes. In addition, the low government spending on healthcare results in high OOP spending and inequitable health care, creating significant disparities between the haves and have-nots.

The current population of Sudan is 45,648,799 as of Thursday, April 7, 2022, based on Worldometer elaboration of the latest United Nations data [5]. Women constitute about 50% of this population [3]. Breast cancer followed by cervical and ovarian cancers are the most common cancers among Sudanese women. According to GLOBOCAN 2020, female age-standardised incidence rates per 100,000 in Sudan were 41.2, 8.7 and 6.7 for breast, cervix and ovary cancer, respectively [2]. However, the GLOBOCAN data is based on Federal Ministry of Health (FMOH) data.

Given that Sudan does not have a functioning national cancer registry, the incidence at a country-wide level is unknown, as most data comes from hospital-based registries [6, 7]. Although some attempts have been made to improve breast cancer awareness and management, less attention is given to cervical and ovarian cancers regarding policy makers’ concerns and resource allocation. Breast cancer early detection campaigns, awareness activities and professional training have been instituted as collaboration between governmental strategies, the private sector, voluntary organisations and the media [6, 8.]. The overall survival of breast cancer patients became comparable to African countries.

Nevertheless, the knowledge, attitude and practice regarding breast cancer screening among Sudanese female workers at secondary-level hospitals are still unsatisfactory [9, 10]. Furthermore, despite proven interventions for primary, secondary and tertiary prevention and the World Health Organization’s (WHO’s) call to action toward eliminating cervical cancer, there has been little progress in addressing the cervical cancer burden in Sudan. Therefore, this short communication intends to shed light on the challenges facing women’s cancers in Sudan, taking cervical cancer as an example.

## The current situation

Outcomes of women’s cancers in general, and cervical cancer in particular, are greatly affected by the fragile Sudanese health system, which is evident throughout the cancer continuum [11]. Moreover, the inexistence of an implemented comprehensive cancer control approach contributes negatively to Sudanese women’s health. Awareness about the Human papillomavirus (HPV), the burden of cervical cancer and its prevention and screening methods is deficient even among highly educated women and nursing students [12, 13]. In addition, public education and youth programmes lack age-appropriate information on known risk factors and preventive practices. The HPV vaccine is yet to be introduced, and Sudan is yet to make use of its eligibility for Gavi support to build an HPV vaccination programme in the country [3, 14]. Therefore, the true prevalence of HPV at the community level has never been studied. As far as the authors are aware, there is no published official documentation of reasons for the lack of access to the HPV vaccine, nor is there a clear endorsed policy as to whether there is a clear plan for introducing the vaccine.

The country has limited screening efforts utilising visual inspection with acetic acid and sometimes Pap smears [15]. However, these efforts are uncoordinated and mostly ineffective as they are not linked to community awareness activities or services to treat premalignant conditions [16].

Given that the cancer stage at diagnosis is a significant predictor of prognosis and survival [17], many hospital-based studies revealed that most patients present in late terminal stages (III & IV), and many die unnoticed or unrecorded [15, 16, 18, 19]. In Sudan, the typical histopathological pattern of cervical cancer is the invasive squamous cell carcinoma; pre-cancerous lesions are seldom seen. A review of cervical lesions diagnosed histologically at the National Health Laboratory revealed that 98.8% of the cervical squamous carcinomas diagnosed in the National Health Laboratory were invasive and 1.2% intraepithelial [20]. The late presentations were associated with older age, African ethnicity, rural areas and lack of insurance, thus demonstrating marked disparities [21].

The delay in accessing cervical cancer care in Sudan is multifactorial, including lack of awareness about the early disease symptoms, cultural factors including fear of stigma, shyness, social beliefs and the enormous dispersion of the population over a vast geographical area [7]. In addition, health system-related factors contribute to difficulties in early access to care and amplify the late presentation. These include a lack of effective screening programmes, the reduced capacity of health care providers at the first point of contact to recognise red flag symptoms and signs of cancer, and the lack of a clearly defined system for a referral from primary to secondary and tertiary levels. Furthermore, the shortage of accessible laboratory and imaging services delays early cancer detection. Finally, appropriate staging protocols based on resources and existing evidence are deficient locally.

The histopathology diagnosis is costly, and the number of active governmental or private sector laboratories providing histopathology services is incompatible with the population size and is not equally distributed. For example, in a country with more than 43 million, there are eleven working public histopathology laboratories at the time of writing this document; seven are located in the capital Khartoum. Additionally, despite social and private Health Insurance in Sudan for 20 years, the country is still far from achieving universal health coverage. Moreover, the sustainability of health insurance is questionable due to low governmental financial resources and lack of affordability by beneficiaries, especially for private Health Insurance [22].

The treatment of invasive diseases, which requires timely access to rapid, stage-appropriate, high-quality, affordable management in the public sector to prevent cancer deaths and enhance health-related quality of life, is severely constrained. Core clinical elements required for managing cervical cancer are cancer surgery, chemotherapy, radiotherapy and palliative care. Surgery, one of the primary modalities for treating early-stage cervical cancer, is challenged by the unavailability of well-equipped operating rooms and gynaecologic oncologists or surgeons specially trained in cervical cancer surgery. There are neither gynaecologic oncologists nor an established training programme for Gynae Oncology currently in Sudan.

Limited availability of radiotherapy is a particularly pressing issue given the primacy of radiotherapy as a treatment modality for cervical cancer. Although the chemo drugs and radiotherapy sessions are free [6], the health system cannot regularly maintain radiotherapy capacity resulting in long waiting lists and frequent treatment breaks, which prolongs the overall treatment time and adversely affects local control [23]. This often results in incomplete therapy because of increasing indirect costs and physical, financial or emotional strain of being far from home for extended treatment periods. Brachytherapy, which maximises survival and local control while minimising late toxicity, suffers periodic unavailability. In Sudan, there were three High Dose Rate Brachytherapy machines in three governmental oncology centres (Khartoum, Medani and Shendi) [24]. At the time of writing this document, the three governmental machines are out of work, while a fourth machine comes into action in a recently opened private hospital. Concurrent chemo-radiotherapy, which is beneficial across all tumour stages, suffers the periodic unavailability of the chemotherapeutic agents [25].

Palliative care is rarely accessible in Sudan and is generally unavailable in primary care, community or home-based settings. Access to pain killers is fraught with legislative barriers resulting in unnecessary suffering. Access to morphine remains limited, with thousands of patients outside Khartoum and Medani having no access to oral morphine [26]. Women with cancer have no access to psycho-oncology, oncology nursing, nutrition support and rehabilitation. Coordination among facilities at different levels of care is missing resulting in fragmentation of care and miscommunication among professionals. Multidisciplinary teams that ensure clinical effectiveness are nearly non-existent. Inaccessibility to all these services hampers the efforts to reduce mortality from women’s cancers, posing challenges in meeting the Sustainable Development Goals (SDGs) targets [27] and making the life of the unfortunate woman with cancer and her family/community a misery.

### Challenges in improving outcomes of women’s cancers in Sudan

While Sudan’s efforts to control cancer, in general, are constrained due to inadequate facilities for care delivery and lack of investment in a sustainable workforce, insufficient local data contribute to stakeholder negligence of women’s cancer control in particular [28].

Sudan’s healthcare spending for 2018 was $60 per capita (4.51% of the Gross Domestic Product ‘GDP’), a 68.76% decline from 2017 [29]. Scientific Research spending is also limited in Sudan. The Ministry of Higher Education and Scientific Research allocated 1 million Sudanese pounds (about 779.000 USD) for all research in 2020. However, this sum rapidly diminished in actual value within a few months because of astronomical inflation and the weakness of the Sudanese pound. In addition, the limited national research funding affected the quantity and quality of the published studies, which are mostly retrospective and institution-based [12]. Despite Sudan’s disadvantage in international financing and partnerships, Sudanese researchers have demonstrated a strong level of local collaboration that is thought to be unusual for a developing country [30]. Several reasons could be hypothesised, including relative isolation secondary to prolonged sanctions, which may have fostered a local gaze research lens with the paradoxical advantage of prioritising local research agenda and collaboration. This can be built upon further to strengthen research on women’s cancers in Sudan.

Sudan has been at a massive disadvantage in international funding and partnerships. Reasons include prolonged sanctions, prolonged periods of dictatorship resulting in political instability, internal conflicts/displacement and lack of affiliation with East African countries at the WHO level. Sudan is bordered by seven countries, five of which are Sub-Saharan African countries with fragile health systems and internal conflicts. In addition to similar epidemiologic and economic profiles, Sudan is heavily entangled with East and sub-Saharan African countries through population movement, including patients and refugees. Despite internal problems, including internal displacement, Sudan is one of the biggest recipients of refugees. It hosts refugees from the Central African Republic, Eritrea, Ethiopia, Chad, South Sudan, the Syrian Arab Republic and Yemen, among other countries [32]. Women constitute one of the most vulnerable populations, and women with cancers in internally displaced and refugee populations have the poorest access to care and worst outcomes. There has been significant interest in cancer in conflict zones, but cancer in people affected by conflict in Sudan has received minimal governmental attention and international attention. Sudan is significantly disadvantaged by not fully aligning with Sub-Saharan Africa, particularly East Africa, given similar epidemiologic and economic conditions. Sudan is a leading Intergovernmental Authority on Development (IGAD) member. This regional political body includes Djibouti, Ethiopia, Kenya, Somalia, South Sudan, Sudan and Uganda. It is considered the first body to address contemporary development, environmental and peace issues in the horn of Africa [33].

Sudan is a member of the WHO Regional Office for the Eastern Mediterranean (WHO EMRO) rather than the WHO Regional Office for Africa (WHO AFRO). Many Sudanese health experts believe that this fragmentation and discordance of Sudan’s geographical and epidemiologic profile with its affiliation with the major international and regional health organisations weaken the impact of these organisations in working with local health policymakers and institutions.

Instability at the Sudan FMOH during the recent transition to democracy and the current return to dictatorship is a substantial hindrance. In addition, observable rapid turnover of the administrative personnel and leadership coupled with weak connections to academia and research institutions constitutes a significant cause of delay in decision-making and strategic planning. For example, the government established a National Strategy for Reproductive Cancers 2 years ago that is yet to be completed.

The lack of national strategies, protocols and programmes challenges Sudan’s sustainable health care system. In addition, most initiatives are started by highly motivated academic advocates, who receive very little support from their educational institutions or the governmental authorities, resulting in a real risk of burnout and external and internal brain drain.

### Opportunities for improving outcomes of women’s cancers in Sudan

Despite all these challenges, there have been significant successes and emerging opportunities. For example, a recent commitment from the Mother and Child Health Department, under the Primary Health Care Directorate in the FMOH, to issue a national strategy to reduce the burden of reproductive cancers. In addition, assigning a unified entity for women’s cancers at the FMOH level will cut short the lengthy policy development and implantation processes.

Networking to bring alignment between the FMOH and other institutions is an opportunity to alleviate the cancer burden on women. Alliances with higher education research institutions to set priorities in research and training is an important achievable advance. Attempts at strengthening public health institutions and their role in professional training, such as medicine, nursing and midwifery, to address population health concerns (social accountability) are expected to help address workforce shortages. Furthermore, collaboration between public and private sectors through memorandums of understanding will help address some of the challenges in access to care. Finally, we should stress the coordinated involvement of several local non-governmental organisations (NGOs) in raising community awareness about cancer screening and early detection.

Cancer detection at a potentially curable stage will improve survival and quality of life. Several interventions could address late-stage presentation and identify the disease at the earliest opportunity. These include universal access to care points ready for prompt early diagnosis of cancer, referral for investigation of ‘Red flag’ symptoms to secondary care and an intact link to diagnosis and treatment without delay.

Decentralising oncology services would reduce the inequity in health care provision in rural areas and remote parts of the country. Sudan has made significant strides in decentralisation. There were only two major oncology centres (Khartoum Oncology Hospital, formerly the Radiation and Isotope Center-Khartoum, and the National Cancer Institute in Wad Medani, Gezira state). Currently, there are ten governmental oncology centres of various capacities in different Sudanese provinces, helping to reduce patient travel and improving local access. Although radiotherapy is provided only in three oncology centres, histopathology and imaging services need more reorganisation.

Sudanese women with cervical cancer will benefit from the national and decentralised adoption of the essential package of palliative care for cervical cancer with its four interventions of medicines, simple equipment, social support in addition to trained human resources [33]

Another promising prospect is the engagement of the diaspora, such as the Sudanese American Physician Association in the Sudan Medical Specialization Board, the main authorized body for postgraduate training and expansion of capacity to respond to healthcare needs in Sudan. This is expected to address numerical and quality gaps in different speciality training and impart lasting capabilities that lead to sustainability [34].

Individual initiatives and efforts in advocacy must be amplified, supported and scaled up. One example is the Breast Cancer Multidisciplinary Team Clinic, established in Khartoum Teaching Hospital. In addition, a weekly multidisciplinary gynae-oncology clinic has preceded it at the Oncology Department of the National Cancer Institute-University of Gezira [24].

Another outstanding effort is the proposed Women’s Cancer Control Center, upgrading the services delivered by the Cervical Cancer Prevention & Research (CCPR) unit, established at Soba University Hospital in August 2018. The team was supported by the Sudanese American Medical Association and the Italian Agency for Development Cooperation. The 3 years’ experience at CCPR clearly illustrated that efforts at screening would undoubtedly be limited by the number of providers trained to treat pre-cancerous lesions. Furthermore, expanding cervical cancer screening should identify asymptomatic invasive cancer cases and will result in more women being diagnosed with early-stage diseases who will need radical surgery aiming for a cure. The CCPR unit experience also confirmed that timely assessment and treatment of women with suspected or confirmed cervical cancer are crucial for saving lives and preventing suffering. Hence, a fellowship in surgical gynaecologic oncology education is highly needed. The training can consider North-South partnerships seeking collaboration with the International Gynecological Cancer Society, similar to successes in Zambia and many African countries [35].

The CCPR unit plans to establish a Comprehensive Women’s Cancer Center of Excellence that implements integrated, evidence-based interventions through the disease continuum. It incorporates all gynaecological cancers, and breast cancer uses the resources. Comprehensive management of invasive women’s cancer requires appropriately qualified health providers in a well-equipped set-up with access to highly functional surgical infrastructure, radiotherapy -chemotherapy services, pathology and imaging. A good base for most of these services is already present in Soba University Hospital, making the enterprise achievable.

Another multidisciplinary approach to tackling women’s cancers is the establishment of the Cervical Cancer in Sudan Research Group. We aim to provide evidence on contextually relevant interventions to improve cancer care, emphasizing down-staging disease and increasing awareness of cervical cancer, especially among healthcare workers. It is a multidisciplinary group with more than 70 participants representing different higher education institutes, Ministry of Health, NGOs and international collaborators. It took an inventory of human resources, equipment and unmet needs. Its overarching goal is to support the stakeholders in establishing health policies and programmes that facilitate preventing and controlling cervical cancer and its early diagnosis, prognosis and management. It will tackle the condition with a holistic approach, integrating different specialities. The collaborative work done by the Ethiopian Cancer Group is a good example [36]. Sudan can also benefit from South-South collaboration and knowledge transfer in emulating the successful adoption of HPV vaccination, similar to Senegal, a predominantly Muslim sub-Saharan country, which introduced it in 2018 in partnership with Gavi [14].

We believe creating a multidisciplinary community of practice for women’s cancers is an excellent opportunity; informal, harnessing all talents to mobilise and support those on the frontline. Additionally, raising awareness and introducing early detection programmes are critical for the better survival of these patients [37]. Furthermore, it is crucial to produce contextual scientific evidence on women’s cancer to inform the national strategic plan. Therefore, recent population-based studies are highly recommended. Developing population-based cancer and death registries to determine disease prevalence and generate reliable death notification protocols is the first step to launching cancer control programmes. Policies are also needed in collaboration with the international community in addressing the cancer care needs of internally displaced and refugee women in Sudan. Novel approaches implemented in other parts of African conflict zones can potentially serve as models for interventions in these vulnerable populations [38, 39].

## Conclusions and the way forward

Women’s cancers in Sudan remain a neglected public health problem, especially cervical cancer. With no current programme for HPV vaccination nor coordinated screening and cancer management efforts, Sudan remains significantly behind in achieving the global strategic goals by 2030 to be on the path to cervical cancer elimination.

Sudan’s government should urgently implement a broad public health strategy to improve outcomes for women with cancer. The cancer control plan should be aligned with international, evidence-based recommendations and adapted to the local circumstances. It should strengthen health literacy, augment different health care interventions, including vaccination, committed screening programmes, early detection and proper diagnosis of symptomatic cases, a programmatic approach to active management and palliative care and ensure robust referral pathways. Services must be comprehensive and coordinated at each step. The attempt should consider overcoming the existing challenges and making the most available opportunities. Other strategies should include fostering research and strengthening collaboration between stakeholders, especially between academia and MOH. In addition, Sudan must forge more effective relationships with international health agencies that align with the epidemiology of disease and Sudan’s geopolitical situation.

## List of abbreviations

CCPR, Cervical Cancer Prevention & Research; FOMH, Federal Ministry of Health; Gavi, Global Alliance for Vaccines and Immunization; HPV, Human papilloma virus; NGOs, Non-governmental organisations; WHO, World Health Organization.

## Conflicts of interest

The authors declare that they have no conflicts of interest.

## Availability of data and materials

Not applicable.

## Funding

This manuscript has not been funded.

## Authors’ contributions

All the authors participated actively in the preparation of the manuscript.
